# Supplementary material for: Deletion of wfs1 Impairs Oligodendrocyte Precursor Cells Dorsal Distribution and Myelination Through the wfs1-hmgcs1 Axis in Zebrafish
Source: Biology (Basel). 2026 Aug 16;15(16):1402. doi: 10.3390/biology15161402 (PMC13510151; doi:10.3390/biology15161402)
Supplement: Supplementary file 1 [file biology-15-01402-s001.zip › File S1-Transcriptome sequencing data and differentially expressed genes (DEGs) in wfs1-- zebrafish at 5 dpf/2.KEGG/wfs1KOvsWT/ALL/src/dre04620.html]

dre04620


- dre:562552

- Up regulated genes

562552(1.50682290794657)

- dre:100002946

- Down regulated genes

100002946(-1.40391573648291)

- dre:792699

- Down regulated genes

792699(-1.84389282152821)

- dre:368481

- Down regulated genes

368481(-2.13889418398421)

- dre:403131

- Down regulated genes

403131(-4.42862381698358)

- dre:561021

- Up regulated genes

561021(2.14284154145004)

- Down regulated genes

561021\_1(-4.68369619674599)

- dre:100000252

- Up regulated genes

100000252\_3(1.94434154854557) 100000252(1.09163460881879)

- Down regulated genes

100000252\_1(-1.7718817168434)

- dre:394198

- Up regulated genes

394198(1.20861445776772)
- dre:570238

- Up regulated genes

570238(2.66701017363204)

Close
